# Krüppel-Like Factors in Metabolic Homeostasis and Cardiometabolic Disease

**DOI:** 10.3389/fcvm.2018.00069

**Published:** 2018-06-11

**Authors:** Yumiko Oishi, Ichiro Manabe

**Affiliations:** ^1^Department of Biochemistry & Molecular Biology, Nippon Medical School, Tokyo, Japan; ^2^Department of Disease Biology and Molecular Medicine, Graduate School of Medicine, Chiba University, Chiba, Japan

**Keywords:** KLF, metabolism, liver, muscle, macrophage

## Abstract

Members of the Krüppel-like factor (KLF) family of transcription factors, which are characterized by the presence of three conserved Cys_2_/His_2_ zinc-fingers in their C-terminal domains, control a wide variety of biological processes. In particular, recent studies have revealed that KLFs play diverse and essential roles in the control of metabolism at the cellular, tissue and systemic levels. In both liver and skeletal muscle, KLFs control glucose, lipid and amino acid metabolism so as to coordinate systemic metabolism in the steady state and in the face of metabolic stresses, such as fasting. The functions of KLFs within metabolic tissues are also important contributors to the responses to injury and inflammation within those tissues. KLFs also control the function of immune cells, such as macrophages, which are involved in the inflammatory processes underlying both cardiovascular and metabolic diseases. This review focuses mainly on the physiological and pathological functions of KLFs in the liver and skeletal muscle. The involvement of KLFs in inflammation in these tissues is also summarized. We then discuss the implications of KLFs' control of metabolism and inflammation in cardiometabolic diseases.

## Introduction

**Figure 1:**
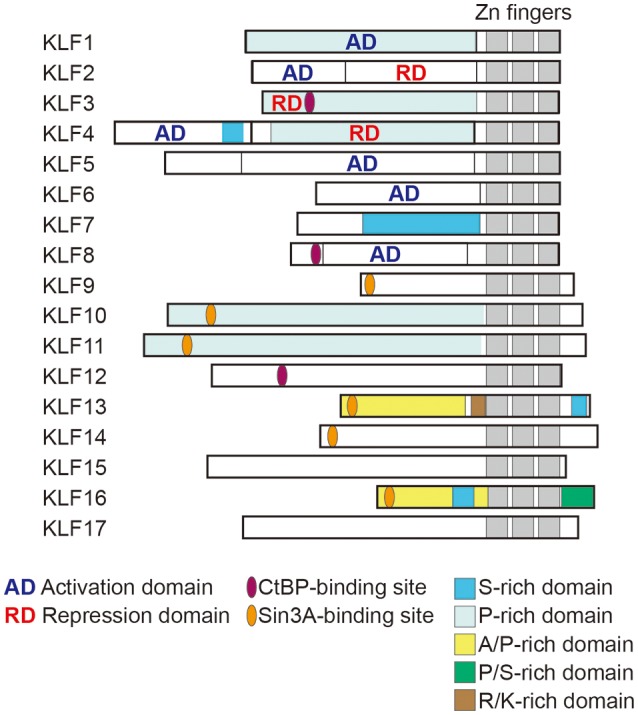
Structure of human KLF proteins. All KLF members have homologous C-terminal DNA binding domains that are composed of three C2H2 zinc fingers. By contrast, their N terminal regions are highly diverse and may contain activation and repression domains and various protein interaction domains. For instance, KLF3, 8 and 12 all contain CtBP-binding motifs, while KLF9, 13, 14, and 16 all contain Sin3A-binding motifs. *KLF18* gene is closely related to *KLF17*, though *KLF18* may not be expressed (pseudogene) (3). Modified from Kim et al. (4).

The Krüppel-like factors (KLFs) belong to a family of zinc-finger containing transcription factors. KLFs regulate diverse biological processes in mammalian tissues, including cell proliferation, differentiation and survival, and tissue development. KLFs are also crucially involved in the maintenance of systemic and tissue homeostasis (1, 2). To date, 18 KLFs have been identified, though the *KLF18* gene is likely a pseudogene (3). All KLFs contain three conserved Cys_2_/His_2_ zinc-fingers within their C-terminal domains (Figure 1) and bind to similar consensus sequences (CACCC-, GC-, or GT- box elements) located within the promoters and enhancers of target genes. Despite their binding to similar sequences, their target genes and functions are diverse and highly context-dependent. But what are the mechanisms that enable KLFs to have distinct and context-dependent functions? First, KLF expression is often highly dependent on cellular and environmental contexts, such as cell type and environmental cues. Second, their varied N-terminal sequences, interactions with other transcription factors and coregulators, and post-translational modifications contribute to the distinct and context-dependent function of each KLF (Figure 1) (4). For instance, it is likely that interactions with other factors and the status of the chromatin opening, which is also context-dependent, limit a KLF's binding to a small subset among the numerous CACCC motifs within the genome. Although there is much still to be learned about the precise mechanisms underlying their functional diversity, previous studies using genetically engineered mouse and cell models have experimentally demonstrated that each KLF family member has distinct functions. Among these diverse functions, control of metabolism appears to be evolutionally conserved among a number of KLFs (5). For example, two of the three KLFs expressed in *C. elegans* are important for lipid metabolism (6).

Metabolic abnormalities such as obesity and diabetes are the major risks for cardiovascular disease, as multiple pathways link metabolic alterations to cardiovascular disease development. For instance, dyslipidemia, such as high low-density lipoprotein (LDL) cholesterolemia, triggers, and promotes atherogenic processes within the arterial wall. Insulin resistance is also involved in vascular pathology and heart failure (7). Mediators secreted from metabolic tissues, including adipokines, myokines, and hepatokines from fat, muscle and liver, respectively, have all been shown to affect cardiometabolic pathologies (8).

Chronic inflammation is another key mechanism that links cardiovascular and metabolic diseases (9). Chronic inflammation is not only a common and pivotal mechanism underlying the initiation and development of both cardiovascular diseases, such as atherosclerosis, and metabolic diseases, such as non-alcoholic fatty liver disease (NAFLD) and diabetes, it also mechanistically connects these diseases. One example is the impact of adipose tissue inflammation on cardiometabolic disease. Visceral obesity induces chronic inflammation within visceral adipose tissue, which may promote cardiometabolic pathology, such as atherosclerosis and type 2 diabetes, in part by promoting inflammation within the affected tissue through release of inflammatory cytokines and free fatty acids from the inflamed adipose tissue (9, 10). Several clinical trials of drugs targeting inflammatory pathways have highlighted the pathological and therapeutic importance of inflammation in cardiometabolic disease (11–13).

In this review, we focus on the roles of KLFs in metabolic regulation and their contributions to metabolic and cardiovascular diseases. Our particular focus is on the physiological and pathological functions of KLFs in the liver and skeletal muscle because these organs are the major sites of regulation of systemic metabolism, and metabolic defects in these organs are essential to type 2 diabetes (14). We will also discuss the impact of KLF regulation in these organs from the perspective of the metabolic modulation and inflammation that promote cardiometabolic disease. Although we do not cover it here, adipose tissue also plays a key role in systemic metabolic regulation and is a pivotal contributor to the development of cardiometabolic disease. Readers are referred to recent reviews on the functions of KLFs in white and brown adipose tissue (15, 16).

## KLFs in liver biology and pathobiology

### KLF6 is essential for liver development

KLF6 plays a central role in fetal liver development (17). Liver organogenesis in mammals depends on vascular and hematopoietic development (18, 19). In mice, for example, systemic deletion of *Klf6* is lethal by embryonic day (E) 12.5, and is associated with markedly reduced hematopoiesis, poorly organized yolk sac vascularization, and an apparent lack of the liver. Consistent with the phenotype in early embryos, *Klf6*^−/−^ embryonic stem (ES) cells display hematopoietic defects following differentiation into embryoid bodies. Moreover, *Klf6*^−/−^ ES cells fail to differentiate into hepatocytes (20). Deletion of *Klf6* in zebrafish independently confirmed that KLF6 is essential for development of endoderm-derived organs, including liver.

### KLFs control glucose and amino acid metabolism in the liver

The liver is a major site of insulin action in adults, as glucose uptake, glycolytic metabolism, and gluconeogenesis all occur there. KLF15 is abundantly expressed in the liver, and its expression is increased by food deprivation and is reduced by feeding. KLF15 regulates gluconeogenesis-related genes such as *Pck1*, which encodes phosphoenolpyruvate carboxykinase (PEPCK) in mice (21). Genetic deletion of *Klf15* results in fasting hypoglycemia due to abnormal gluconeogenesis and defects in the use of amino acids as sources of gluconeogenic substrates. *Klf15* deletion also decreases hepatic expression of genes encoding gluconeogenic and amino acid catabolic enzymes (22, 23). For instance, in the *Klf15*^−/−^ liver, reductions were observed in the expression of alanine aminotransferase 1 (*Alt1*), proline dehydrogenase (*Prodh*), tryptophan 2,3-dioxygenase (*Tdo2*), and 4-hydroxyphenylpyruvic acid dehydrogenase (*Hpd*) genes, which catabolize alanine, proline, tryptophan, and tyrosine, respectively (23). Alanine aminotransferase catalyzes conversion of alanine to the gluconeogenic substrate, pyruvate. As expected, the ability to utilize exogenous alanine for glucose production is disrupted in *Klf15*^−/−^ mice. These findings demonstrate that KLF15 as an important regulator of gluconeogenesis and amino acid catabolism in the liver (Figure 1). Interestingly, KLF15 is involved in the action of metformin, one of the first-line medications for the treatment of type 2 diabetes (23). Metformin suppresses gluconeogenesis in hepatocytes by reducing KLF15 levels through enhancement of its ubiquitination and degradation and downregulation of its mRNA.

KLF6 is also involved in glucose metabolism in the liver. KLF6 transactivates *GCK*, which encodes glucokinase, a rate-limiting enzyme for hepatic glucose utilization and a major regulator of blood glucose homeostasis (24). In liver tissue from humans with NAFLD, expression of the full-length KLF6 isoform and glucokinase are correlated, suggesting KLF6 regulates *GCK* under pathological conditions in the liver. Glucokinase facilitates post-prandial extraction of blood glucose by liver by promoting glucose storage as glycogen through glycogenesis or as lipid through lipogenesis (25, 26). Indeed, hepatic overexpression of *GCK* in rodents reduces blood glucose and induces hypertriglyceridemia and hepatic steatosis. In addition, in humans *GCK* mRNA levels were associated with markers of de novo lipogenesis and the triglyceride content of liver tissues over a wide range of steatosis levels (27). Also reported, however, were conflicting data indicating that *KLF6* and *GCK* mRNA levels were downregulated in advanced steatosis as compared with mild steatosis (24). Although the reason for the conflicting observations is not immediately clear, expression of *KLF6* and *GCK* may be differentially regulated in mild vs. advanced steatosis. Nevertheless, these findings suggest KLF6 is involved in the development of NAFLD, in part by controlling *GCK* expression. However, there is a need of further analysis of its pathological actions and regulation at different stages of the progression of NAFLD, and we will discuss its contribution to non-alcoholic steatohepatitis (NASH) development in the following section.

### KLFs control lipid metabolism in the liver

Regulation of lipid metabolism is another important function of the adult liver. In addition to its functions in glucose metabolism, KLF15 also contributes to hepatic lipid metabolism. *Klf15* deletion ameliorates hepatic insulin resistance induced by a high-fat diet (HFD) without affecting the endoplasmic reticulum (ER) stress or hepatic inflammatory responses that typically accompany insulin resistance (28). Adenovirus-mediated hepatic *Klf15* knockdown or systemic *Klf15* deletion increases hepatic levels of ER stress markers in mice fed with a HFD. Interestingly, HFD-induced systemic and hepatic insulin resistance is ameliorated by genetic interventions targeting *Klf15*. Moreover, while an ER stress activator, tunicamycin, induces liver steatosis and insulin resistance, these hepatic responses are much reduced in *Klf15*^−/−^ mice. By contrast, *Klf15* inhibition increases JNK phosphorylation and proinflammatory cytokine expression. Thus, *Klf15* deletion uncouples hepatic insulin resistance and steatosis from ER stress and inflammation in HFD-induced obesity. Although the precise mechanism remains unknown, Jung et al. proposed that KLF15 activates mTORC1 signaling. In *Klf15*^−/−^ mice, enhanced fatty acid oxidation presumably due in part to inhibition of mTORC1 may protect the liver from steatosis (29, 30). These observations suggest KLF15 is a crucial contributor to the regulation of hepatic metabolism, and its perturbation leads to alterations in HFD-induced hepatic pathology.

Takeuchi et al. very recently showed that KLF15 plays an important role in the switching between lipogenesis and gluconeogenesis during fasting (31). Through a series of experiments examining *in vivo* promoter activity, these investigators identified KLF15 as a regulator of *Srebf1*, which encodes SREBP-1, a transcription factor that controls cellular lipid metabolism. One of the two SREBP-1 isoforms, SREBP-1c, is particularly involved in controlling genes required for lipogenesis, including *Fasn* (fatty acid synthase) and *Acc* (acetyl-CoA carboxylase) (32). Fasting markedly reduces hepatic expression of *Srebf1*, while *Klf15* expression is increased. During fasting, upregulated KLF15 interferes with LXR/RXR-dependent transactivation of *Srebf1* transcription by forming a complex with LXR/RXR and a corepressor RIP140 (31). Because KLF15 promotes gluconeogenesis during fasting (see above), induction of KLF15 through fasting rapidly switches hepatic metabolism from lipogenesis in the fed state to gluconeogenesis in the fasting state.

### KLFs contribute to NAFLD development

In addition to its essential role in fetal liver development, KLF6 is involved in the pathogenesis of liver steatosis and fibrosis (33). Expression of KLF6 is reportedly increased during early hepatic fibrosis in response to liver injury induced by CCl_4_ administration in rats (34). KLF6, which is induced early after injury, transcriptionally activates transforming growth factor β1 (TGF-β1) and TGF-β receptors in hepatic stellate cells (35). Activation of TGF-β signaling in stellates cells upregulates genes involved in fibrosis, such as those encoding extracellular matrix, plasminogen activator inhibitor and platelet-derived growth factor receptors (33). An association between TGF-β1 and liver fibrosis was confirmed by the observation that liver-specific, forced expression of TGF-β1 induces liver fibrosis associated with increased expression of collagen I in hepatic stellate cells (36).

Liver steatosis is frequently observed in heavy drinkers but also in conditions such as obesity, diabetes, and dyslipidemia. Non-alcoholic fatty liver is one of the two types of NAFLD and can progress into NASH, which is characterized by inflammation and fibrosis in addition to steatosis. *Klf6* and *Tgfb1* are upregulated during the progression of rat models of NASH (37), and hepatocyte-specific *Klf6* deletion is protective against HFD-induced liver steatosis and insulin resistance, which suggests KLF6 contributes to NASH development (38). KLF6 also acts at the post-transcriptional level to upregulate peroxisome proliferator-activated receptor α (PPARα) expression. PPARα controls several genes that promote insulin resistance and NASH, including *Trib3*, whose expression is correlated with that of *KLF6* in liver tissue from humans with NAFLD (38). However, the actions of PPARα in the context of NASH are much more complex. For instance, while *Ppara* deletion reportedly enhances HFD-induced NASH pathology (39), a conflicting observation has also been reported (40). In line with the anti-fibrotic function of PPARα, a recent whole genome profiling study identified pro-fibrogenic dermatopontin (encoded by *Dpt*) as a potential downstream mediator of KLF6 in NASH (41). PPARα activation reduced *Dpt* expression, possibly by decreasing *Klf6* and *Tgfb1* expression. KLF6 thus appears to be profibrogenic in the liver, but its interactions with PPARα are complex, and their effects in NASH may be context-dependent.

Hepatic *KLF6* expression is associated with more advanced stages of NAFLD in humans (42), and the presence of KLF6 splicing variants that antagonize the full-length form increases the complexity of its role in the ailment (43). A single nucleotide polymorphism (*KLF6–IVS1-27G*>*A*, rs3750861) that promotes alternative splicing of KLF6 into the dominant-negative variant was shown to negatively associate with the level of NASH-related fibrosis (42), supporting the notion that full-length KLF6 promotes fibrosis. But although both experimental and clinical findings suggest KLF6 promotes NASH, KLF6 levels correlate negatively with steatosis levels in NAFLD samples (24). This suggests KLF6 may have different functions at different stages of NASH development. Because KLF6 also affects stellate cells and macrophages, during NASH development it may be involved in a variety of pathological processes, including hepatocyte metabolism, myofibroblastic activation of stellate cells, fibrosis, and inflammation. Consequently, KLF6's roles during NASH development are likely complex and cell- and disease stage-dependent.

KLF2 is also involved in hepatic steatosis and is significantly elevated in the livers of obese mice. Adenovirus-mediated overexpression of *Klf2* induces accumulation of triglycerides in lean mice, while silencing *Klf2* ameliorates liver steatosis in obese ob/ob mice. KLF2 acts by directly upregulating expression of CD36, which mediates hepatic triglyceride accumulation (44).

### KLFs involved in the regulation of macrophages in liver and artery disease

In addition to hepatocytes, recent studies have revealed the crucial contributions made by non-hepatocytes to the control of metabolism and to pathology in the liver (45, 46). In particular, liver macrophages play critical roles in metabolism, inflammation, fibrosis, and repair. Macrophages exhibit diverse phenotypes and functions in response to environmental cues (47). The M1/M2 classification was established based mainly on observations of cultured macrophages *in vitro* and has been used to group the variety of macrophage phenotypes into two subgroups (48, 49). Exposure to TLR ligand or Th1 cytokines, such as TNF-α, and IFN-γ, activates macrophages into the proinflammatory, M1 phenotype. M1 macrophages express proinflammatory cytokines and reactive oxygen species. By contrast, Th2 cytokines, such as IL-4 and IL-13, induce the M2 phenotype, though other factors are likely involved in *in vivo* settings. M2 macrophages are known to be essential for parasite clearance and have also been shown to promote resolution of inflammation and fibrosis (50, 51).

Although the M1/M2 dichotomy has been widely used, it is now clear that it does not adequately encompass the diversity and plasticity of macrophage phenotypes and functions. For instance, M1 and M2 markers can be expressed simultaneously in macrophages *in vivo* (52). Moreover, macrophages *in vivo* must respond to numerous signals other than the model stimuli used *in vitro* and express very divergent transcriptomes. In addition, while proinflammatory M1 macrophages are often said to promote inflammation and damage tissue, they are also essential for resolution and regeneration in several injury models, such as muscle injury, and regeneration (53). Consistent with that observation, M1 macrophage-derived inflammatory cytokines, such as TNF-α, have been shown to positively regulate healing and regeneration. As such, the simple M1/M2 dichotomy based on expression of a few marker genes cannot predict the functions of macrophages within complex inflammation and healing processes *in vivo*. In that regard, although several KLF members have been shown to be important for M1/M2 activation, mainly in *in vitro* settings, less clear are their regulatory functions during macrophage activation in *in vivo* inflammatory settings.

Several KLF members are known to be involved in the activation of macrophages (Figure 3). KLF4 is important for the IL-4-induced M2 phenotype in macrophages, while deletion of *Klf4* enhances expression of M1 macrophage-related genes, demonstrating that KLF4 promotes M2 polarization of macrophages (54, 55). KLF2 inhibits NF-κB-dependent proinflammatory activation of macrophages (56, 57). By contrast, KLF6 acts cooperatively with NF-κB to promote proinflammatory gene expression and to suppress M2 marker gene expression, thereby promoting M1 polarization (58, 59). KLF6 also promotes macrophage motility and recruitment, in part by suppressing BCL6 (60). These results suggest KLF6 promotes proinflammatory activation of macrophages. However, myeloid-specific deletion of *Klf6* promoted aortic inflammation in a mouse model of aortic aneurysm (61). In that study, KLF6 negatively regulated production of GM-CSF, which is critical for aneurysm development and inflammation. These conflicting findings highlight the need for more studies of macrophage KLF functions in *in vivo* inflammatory models.

Myeloid-specific *Klf4* deletion augments HFD-induced obesity, insulin resistance and adipose tissue inflammation (54), which is indicative of the systemic metabolic impact of KLF4-dependent regulation of macrophage activation. In addition, a pivotal contribution of KLF4-dependent regulation of macrophage function to cardiovascular disease is exemplified by the observation that myeloid *Klf4* deficiency augments vascular inflammation and atherosclerotic lesion formation in *Apoe*^−/−^ mice (62). A surprising function of KLF4 in atherosclerosis was also recently reported (63). KLF4 promotes phenotypic modulation of smooth muscle cells (SMCs) from a highly differentiated state to a less differentiated “synthetic” phenotype. Lineage tracing studies identified phenotypically modulated SMCs that express macrophage markers within atherosclerotic plaques, indicating that SMCs are able change their phenotype, even becoming macrophage-like. KLF4 appears to regulate this transition to a macrophage-like phenotype. Collectively then, whereas KLF4 in myeloid-lineage cells appears to be atheroprotective, KLF4 in SMCs is proatherogenic. These results point to differential functions of KLF members in different cell types in pathology. Moreover, recent studies also showed that myeloid-lineage cells can acquire SMC-like phenotypes (64, 65). These findings question the lineages of traditionally identified SMCs and macrophages and the functions that have often been assigned to them (e.g., anti- and pro-atherogenic).

### KLF14 regulates reverse cholesterol transport

Within the liver, macrophages modulate hepatocyte metabolism during homeostasis and during development of NAFLD. They also play a key role in the chronic inflammation contributing to NASH. In mouse models of diet-induced NAFLD, the liver-resident macrophages, Kupffer cells, increase hepatic triglyceride accumulation, and proinflammatory cytokine production and suppress fatty acid oxidation and insulin sensitivity (66). HFD-induced obesity also increases numbers of monocyte-derived macrophages in the liver, where they promote inflammation and insulin resistance (67). Han et al. recently showed that a nuclear receptor, retinoic-acid-related orphan receptor α (RORα) induces M2 polarization by activating KLF4 in Kupffer cells, and that myeloid-specific deletion of *Rora* aggravated HFD-induced hepatic steatosis and inflammation (68). Interestingly, M2-polarized Kupffer cells produce IL-10, which reduces lipid accumulation and lipotoxicity in hepatocytes, suggesting liver macrophages control both inflammation and metabolism. In that regard, perturbing M2 activation of Kupffer cells through *Ppard* deletion reportedly impairs hepatic fatty acid oxidation and promotes hepatic steatosis (69), though the precise mechanism by which hepatic metabolism is regulated remains unknown. Macrophages are thus integral to the control of hepatic lipid metabolism, and KLF4 appears to be a critical regulator of these macrophages.

KLF14 expression is reduced in the livers of dyslipidemic model mice, and hepatic overexpression of human *KLF14* increased plasma high density lipoprotein (HDL) cholesterol levels by inducing apolipoprotein A-I (ApoA-I), a major protein component of HDL particles (70). Conversely, loss of *Klf14* decreased HDL cholesterol levels in the liver. KLF14 transactivates hepatic *Apoa1* transcription.

Epidemiological studies have shown an inverse association between plasma HDL cholesterol levels and coronary heart disease (71). ApoA-I is primarily responsible for reverse cholesterol transport (RCT), through which cholesterol is transported from peripheral tissues back to the liver by HDL (72). RCT is thought to be a major mechanism underlying the anti-atherogenic effects of HDL cholesterol. The crucial initial step in RCT from atherosclerotic lesions is efflux of cholesterol from macrophages to extracellular acceptors, namely HDL. Upregulation of hepatic *Klf14* expression may thus protect against atherosclerosis (Figure 1).

Guo et al. used drug screening to identify perhexiline as an activator of *Klf14* expression (70). Perhexiline is a prophylactic antianginal agent thought to act by inhibiting mitochondrial carnitine palmitoyltransferase-1 (CPT-1). Administration of perhexiline increases HDL cholesterol and ApoA-I levels and suppressed atherosclerosis in *Apoe*^−/−^ mice. Because genome-wide association studies have shown that genetic variations near the *Klf14* locus are associated with HDL cholesterol levels, coronary heart disease, and metabolic syndrome (70), therapeutic intervention targeting KLF14 is an attractive strategy for the treatment and prevention of human dyslipidemia and atherosclerosis.

## KLFs in skeletal muscle biology and pathobiology

### KLFs control muscle regeneration and development

Skeletal muscle is the dominant organ for locomotion, postural maintenance, and energy metabolism in mammals. It is the largest organ in non-obese subjects and a major site of insulin- and exercise-stimulated glucose disposal (73, 74), with a remarkable capacity for repair and regeneration in response to injury. During the course of embryonic development, mesenchymal progenitor cells originating from the somites undergo a multistep differentiation process to form skeletal muscle (75). Muscle satellite cells are myogenic precursor cells formed during embryonic development, and are also present in a quiescent state within adult muscle. In response to muscle damage, satellite cells are activated and assume a myoblast identity. Satellite cell-derived myoblasts undergo differentiation and fusion to form myotubes that replace the damaged myofibers (76, 77). The molecular mechanism that controls muscle regeneration recapitulates many aspects of the process of muscle development and many of the transcription factors that control embryonic myogenesis contribute to adult regenerative myogenesis (77, 78).

Skeletal muscle repair is conducted through activation, proliferation, and differentiation of satellite cells, a population of muscle stem cells that reside within a niche between the basal lamina and the sarcolemma of associated muscle fibers. A family of four myogenic regulatory factors (MRFs) govern early skeletal muscle development and also control the postnatal muscle regeneration program (77, 79, 80). These MRFs include the myogenic basic-helix-loop-helix type transcription factors MyoD and Myf5, which bind to regulatory regions of skeletal muscle-specific genes, where they determine myogenic fate and initiate the differentiation cascade (77). Thereafter, MyoD acts in cooperation with myogenin and MRF4 to increase expression of late target genes through a feed-forward mechanism that regulates terminal differentiation (81). MRFs also interact with other transcription factors, including MEF2 in myogenesis (77, 82), and recent studies indicate the involvement of several KLFs.

KLF7 is critical for maintaining satellite cell quiescence *in vitro* through activation of p21 expression (83). KLF7 expression is increased by TGF-β and Notch signaling, which controls satellite cell quiescence and myoblast arrest. KLF7 thus appears to be important for maintenance of satellite cell quiescence, though this needs to be tested in *in vivo* settings.

We found that KLF5 regulates muscle differentiation and regeneration by directly controlling muscle-specific genes in cooperation with MyoD and MEF2 (77). During muscle regeneration after injury caused by cardiotoxin injection, expression of KLF5 is upregulated in the differentiating myoblasts and newly formed myofibers, and the expressed KLF5 is recruited to MyoD binding sites. Interestingly, association of MyoD with its binding sites is greatly reduced in the absence of KLF5, which is consistent with close cooperation between these two transcription factors. Satellite cell-specific *Klf5* deletion using the *Pax7-CreER* line delays and impairs muscle regeneration, confirming the role of KLF5 in muscle repair. Notably, inflammation and fibrosis are enhanced in injured muscle tissues from satellite cell-specific *Klf5*^−/−^ mice (77), which highlights the close interplay between myocyte differentiation and regulation of inflammation during muscle regeneration after injury (84).

Fusion of myoblast into multi-nucleated fibers are necessary for the maturation and differentiation of myotubes. It is reported that KLF2 and KLF4 are upregulated in differentiating muscle cells and promote muscle cell fusion (85). During muscle differentiation of C2C12 myoblasts, *Klf2* and *Klf4* are upregulated by ERK5, a member of extracellular signal-regulated kinase (ERK) family, at least in part through activation of Sp1, which transactivates *Klf2* and *Klf4* transcription. KLF2 and KLF4 in turn transactivate *Npnt*, which encodes nephronectin, a mediator required for muscle fusion. The MEK5-ERK5-Sp1-KLF2/4-nephronectin pathway is thus crucial for muscle cell fusion and myotube differentiation of C2C12 cells, though the function of this pathway remains to be tested *in vivo*.

KLF3 was identified as a factor that enhance muscle creatine kinase expression by binding to a GC-rich sequence in the muscle creatine kinase (*Mck*) promoter, in concert with serum response factor (SRF) (86). In addition, their chromatin immunoprecipitation (ChIP) assays indicated that KLF3 binds to the promoter regions of myosin heavy chain IIa (*Myh2*), *Six4*, skeletal α-actin (*Acta1*), and calcium channel receptor α-1 genes in cultured muscle cells, suggesting KLF3 controls muscle genes, though its actions during muscle development and regeneration have not been directly tested.

Collectively, these results demonstrate that several KLF members play important roles in the differentiation of C2C12 and satellite cells. However, with the exception of KLF5 in muscle regeneration, their *in vivo* functions are less clear. Future studies could further address the functions of KLFs during muscle regeneration and embryonic development by, for example, deleting selected *Klf* s from myogenic precursor cells using *Pax3* and *Pax7* promoter-driven cre lines.

### KLFs control muscle metabolism

KLF15 is a critical regulator of skeletal muscle nutrient catabolism and a key determinant of exercise capacity (22, 87, 88). KLF15 expression is low during development and robustly induced during postnatal maturation (87). Nonetheless, *Klf15* knockdown does not interfere with muscle differentiation of C2C12 cells (89), and the skeletal muscle in *Klf15*^−/−^ mice exhibit no clear developmental defects (87). In addition, KLF15 controls expression of slow-type myosin heavy chain (*Myh7*), suggesting it contributes to myofiber typing (89), though *Klf15*^−/−^ mice exhibit no changes in their fiber type compositions (87).

**Figure 2:**
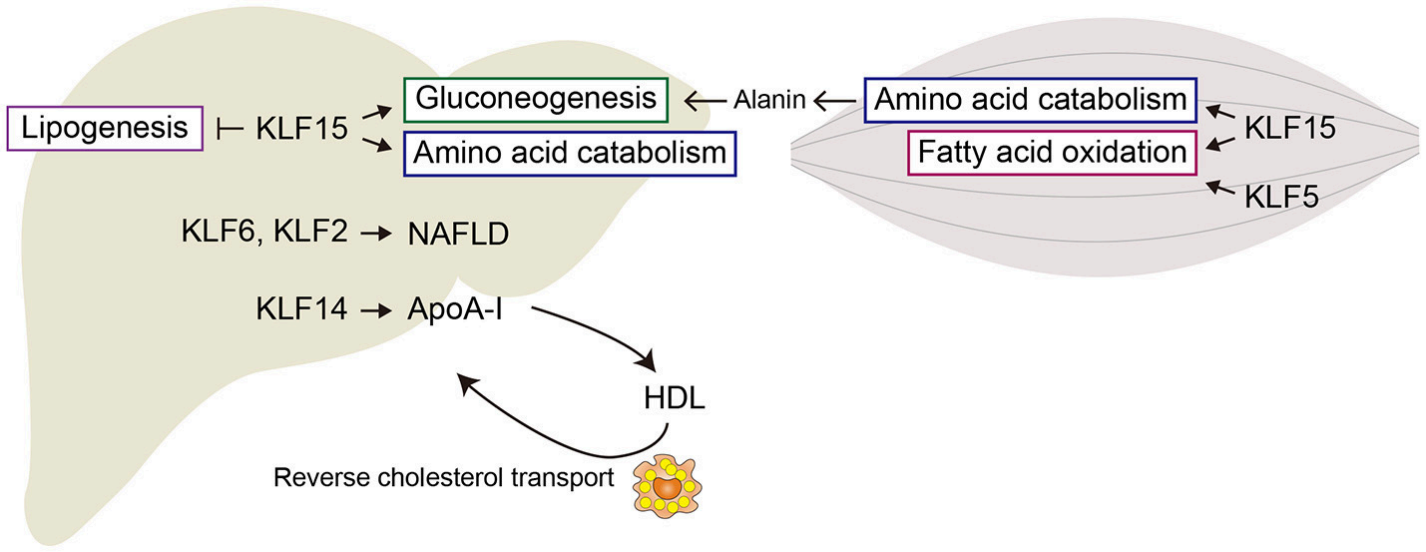
Metabolic regulation by KLFs in liver and skeletal muscle. Shown are major metabolic pathways regulated by KLFs. In liver, KLF15 enhances gluconeogenesis and amino acid metabolism, but inhibits lipogenesis. KLF2 and KLF6 are involved in the development of steatohepatitis and liver fibrosis. In skeletal muscle, KLF15 regulates amino acid catabolism and fatty acid oxidation. KLF5 is also involved in the regulation of fatty acid oxidation.

As in the liver, KLF15 in muscle regulates genes involved in amino acid and lipid metabolism. *Klf15* expression in both skeletal muscle and liver is upregulated by fasting (21, 31, 90). During starvation, amino acids derived from muscle proteins are degraded to ketoacids, which are metabolized to pyruvate by PEPCK and pyruvate kinase. Alanine aminotransferase is active in skeletal muscle, so much of the pyruvate produced is transaminated to alanine. The alanine is then transported to the liver and used for gluconeogenesis (Figure 2). Branched-chain amino acids (BCAAs) are the major donors of amino groups for alanine synthesis (91). In *Klf15*^−/−^ skeletal muscle, expression of *Bcat2*, encoding mitochondrial branched-chain aminotransferase 2, which catalyzes the first step in the oxidation of BCAAs, is downregulated (22). This is consistent with the observation that KLF15 activated during fasting enhances the flux of BCAA-derived carbons into the gluconeogenic pathway. It thus appears that KLF15 acts collaboratively in both the liver and skeletal muscle to promote glucose production in the liver.

**Figure 3:**
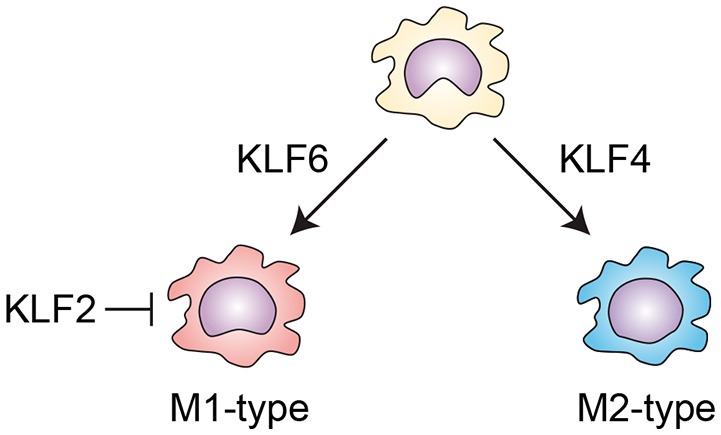
KLFs control macrophage activation. KLF4 is important for the IL-4-induced M2 phenotype in macrophages. By contrast, KLF6 promotes proinflammatory gene expression and suppresses M2 marker gene expression, thereby promoting M1 polarization. KLF2 inhibits NF-κB-dependent proinflammatory activation of macrophages.

*Klf15* is also induced in both mouse and human muscle by acute endurance exercise (87). *Klf15*^−/−^ mice exhibit diminished endurance capacity during treadmill running, which is indicative of the importance of KLF15 for endurance exercise performance. In the absence of KLF15, the soleus muscle (slow twitch muscle), which uses predominantly lipid oxidation for energy production, has reduced capacity for repetitive contraction, whereas fast-twitch function is largely unaffected. In *Klf15*^−/−^ soleus muscle there is dysregulation of genes involved in fatty acid partitioning/transport, fatty acid oxidation and lipid storage. As expected from these alterations, *Klf15*^−/−^ muscle exhibits reduced lipid utilization. These results indicate that KLF15 controls the transition from carbohydrate catabolism to higher reliance on lipid oxidation during endurance exercise (87, 92).

It is previously demonstrated that the involvement of KLF5 in fatty acid metabolism in skeletal muscle (93). We found that heterozygous *Klf5* knockout mice show resistance to diet-induced obesity accompanied by increased systemic energy expenditure. Under basal conditions, SUMOylated KLF5 associates with transcriptionally repressive regulatory complexes containing unliganded PPARδ and co-repressors, which inhibit expression of the lipid oxidation genes *Cpt1b, Ucp2*, and *Ucp3*. Upon agonist stimulation of PPARδ, deSUMOylation of KLF5 is induced and the unSUMOylated KLF5 associates with transcriptional activation complexes containing liganded PPARδ and coactivators, which then transactivates lipid oxidation genes.

Drosatos et al. recently showed that KLF5 directly regulates *Ppara*, which controls genes involved in lipid metabolism in cardiomyocytes (94). Cardiomyocyte-specific *Klf5* deletion induces cardiac dysfunction and lipid accumulation in aging mice. In models of diabetes, cardiac expression levels of *Klf5* and *Ppara* were correlated, suggesting involvement of KLF5 in diabetic cardiomyopathy. While the function of the KLF5-PPARα pathway in skeletal muscle is unknown at the present, these findings suggest that interactions between KLF5 and PPARs are important for regulation of lipid metabolism in muscle.

### KLFs control skeletal muscle growth and pathological wasting

Recent studies suggested KLF15 is involved in regulating muscle mass in both physiological and pathophysiological conditions. Skeletal muscle loss, so called muscle atrophy, occurs under various conditions, including prolonged disuse, sepsis, cachexia, starvation, type 2 diabetes, and aging (95). Muscle mass reflects the dynamic balance between anabolic and catabolic processes, and muscle atrophy in adult tissues occurs when the rate of protein degradation exceeds that of protein synthesis (96). mTORC1 is one of two protein complexes that contain mammalian target of rapamycin (mTOR), which controls protein synthesis in response to growth factors, energy status, oxygen, and amino acids, especially BCAAs (97). Protein degradation is primarily mediated via two major pathways: the ubiquitin-proteasomal pathway and autophagic/lysosomal pathway (96).

Glucocorticoids affect metabolism in skeletal muscle, and the effect of their prolonged use is muscle atrophy. Glucocorticoid (dexamethasone) induces *Klf15* as well as atrophy-related genes (collectively termed “atrogenes”) such as *Fbxo32* (atrogin-1), *Trim36* (MuRF1), *Foxo1* (FoxO1), and *Mstn* (myostatin) in muscle, but does not affect *Klf15* expression in the liver. Glucocorticoid-induced *Klf15* expression is mediated by direct transactivation of *Klf15* transcription by glucocorticoid receptor (GR) (88). KLF15 transactivates *Fbox32* and *Trim36* expression in cooperation with FoxO1 in response to dexamethasone, and the adenovirus-mediated overexpression of *Klf15* induces expression of atrogenes (88), although conflicting data have been reported (98). As with fasting, KLF15 transactivates *Bcat2* transcription and enhances BCAA catabolism in response to dexamethasone. Amino acids, particularly BCAAs, activate mTOR, while overexpression of KLF15 suppresses mTOR activity and causes muscle atrophy. It thus appears that KLF15 acts in concert with other mediators to control expression of atrogenes and BCAA degradation in response to prolonged use of glucocorticoids. The GR-KLF15 axis promotes muscle breakdown and nutrient transfer from muscle to the liver under stressful conditions associated with excess levels of glucocorticoids. However, chronic dexamethasone induces muscle atrophy in *Klf15*^−/−^ as well as wild-type mice (98), which means chronic dexamethasone can also induce muscle atrophy independently of KLF15. Moreover, while adenovirus-mediated strong overexpression of *Klf15* in muscle induces expression of atrogenes (88), dexamethasone-induced upregulation of atrogenes is not affected by deletion of *Klf15* (98). Furthermore, moderate overexpression of *Klf15* in skeletal muscle via a transgene did not induce atrogenes or muscle atrophy (98). Consequently, KLF15's role in atrogene expression remains unclear. Further studies will be needed to clarify the functional involvement of KLF15 in muscle wasting, though results of earlier studies suggest KLF15 may control muscle metabolism in both physiological and pathological settings.

Whereas excessive or sustained glucocorticoid exposure induces muscle atrophy, moderate or transient use of glucocorticoid enhances muscle performance in both animals and humans (98). Moreover, increases in endurance exercise capacity were observed in wild-type mice receiving a single dose of dexamethasone, but no such increase was observed in *Klf15*^−/−^ mice. Comparing transcriptomes in muscle tissues from wild-type and *Klf15*^−/−^ mice treated with a single dose of dexamethasone, Morrison-Nozik et al. found that KLF15 is important for induction of genes related to amino acid and lipid metabolism, but not atrophy-related genes (98). It appears that low-dose and/or transient glucocorticoid induces KLF15 that controls metabolic genes without inducing atrogenes. A separate study comparing the effects of daily and weekly administrations of glucocorticoid found that *Klf15* expression is increased by weekly administration of glucocorticoid, but not by daily administration (99). Interestingly, histone marks at the GR binding site on *Klf15* indicate that while weekly glucocorticoid activate the enhancer, a daily regimen inhibited it, despite strong recruitment of GR to the region. This suggests GR exerts highly context-dependent regulatory effects on *Klf15* and muscle gene transcription.

The physiological importance of KLF15 is also suggested in the context of muscular dystrophy and muscle repair. *Klf15* deletion exacerbates the dystrophic phenotype in *mdx* mice, a model of Duchenne muscular dystrophy, while 5-fold overexpression of *Klf15* ameliorates the phenotype (98). Low-dose or weekly administration of glucocorticoid suppresses the dystrophic phenotype, while daily glucocorticoid promotes muscle atrophy in *mdx* mice (98, 99). A weekly regimen of glucocorticoid, but not a daily regimen, also improves muscle repair following cardiotoxin-induced acute muscle injury (99). It appears that KLF15 is involved in maintaining muscle physiology in response to a variety of stresses, including exercise, muscle injury, fasting, and glucocorticoid, presumably through regulation of muscle metabolism. However, excessive activation or dysregulation of KLF15 may lead to muscle wasting in response to reduced mTOR activity due to altered BCAA metabolism and possibly induction of atrophy-related genes, though its pathological mechanism needs to be further analyzed.

A recent study showed that KLF15 also critically controls metabolic genes in the heart (100). *Klf15* deficiency alters the circadian oscillation of the expression of a large number of genes, particularly genes related to metabolism. KLF15 appears to regulate genes involved in lipid and amino acid catabolism, presumably to support ATP production during the active phase. Based on the effects of cardiomyocyte-specific disruption of the cellular clock, Van Laake et al. proposed that “the cardiomyocyte clock promotes oxidative metabolism at the sleep-wake transition (in anticipation of increased energetic demand upon awakening), augments nutrient storage toward the end of the awake period (in anticipation of the upcoming fast during the sleep period), and increases cellular constituent turnover at the beginning of the sleep period (facilitating repair/renewal of the myocardium prior to awakening)” (101). Accordingly, the circadian control of cardiac metabolism by KFL15 may also regulate the shift toward cardiac remodeling and repair during the inactive phase by regulating metabolism as well as the genes involved in remodeling and growth (100, 102). In addition to physiological cardiovascular regulation (e.g., heart rate and blood pressure), time-of-day-dependent changes are also observed in diseases (e.g., arrhythmia, sudden cardiac death, and myocardial infarction). In that regard, KLF15 controls diurnal expression of Kv channel interacting protein 2 (KChIP2), a critical subunit required for generating the transient outward potassium current (103). *Klf15* deletion causes loss of diurnal QT variation, abnormal repolarization, and greater susceptibility to ventricular arrhythmias. It thus appears KLF15 is a key regulator of time-dependent cardiac physiological and pathological events.

Systemic deletion of *Klf10* results in hyperplasia and hypertrophy of both slow (soleus) and fast (extensor digitorum longus) muscles and leads to glycolytic hypertrophy in mice, regardless of muscle type (104). In addition, overexpression of *Klf10* suppresses differentiation of C2C12 myoblasts (105). *Klf10* is induced by TGF-β signaling, and KLF10 expression is upregulated by myostatin, a member of TGF-β family that inhibits myogenesis. These findings suggest KLF10 acts as a mediator of myostatin's negative effects on myogenesis and muscle growth, though this idea needs to be directly tested in *in vivo* settings.

## Conclusion and future perspectives

KLFs control a variety of processes in the liver and skeletal muscle (Table 1). In addition to their roles in development, their functions in the control of metabolism are essential for both local tissue and systemic metabolic homeostasis in the context of dynamic variation in the supply of and demand for energy. In that regard, KLF15 is well-studied for its coordinated actions in the maintenance of systemic metabolism. During fasting, KLF15-mediated upregulation of BCAA metabolism in skeletal muscle and upregulation of gluconeogenesis in the liver coordinate the flux of substrates for gluconeogenesis from muscle to liver to maintain blood glucose levels (106). The coordination of KLF15 function in metabolism is also suggested by its involvement in the circadian regulation of nitrogen (107). In *Klf15*^−/−^ mice, the circadian rhythmicity in circulating amino acid and urea levels is markedly altered. That *Klf15* expression exhibits circadian rhythm in both liver and muscle suggests KLF15 coordinately controls oscillatory amino acid metabolism. These findings have established KLF15 as a critical physiological regulator of liver and muscle metabolism. Accordingly, dysregulation of KLF15 may contribute to liver and muscular pathologies by affecting metabolism. Similarly, alterations in metabolism resulting from dysregulation of other KLFs may be involved in the development of metabolic disease, though this idea remains largely untested.

**Table 1:** Functions of KLFs in liver, skeletal muscle, and macrophages.

	**Liver**	**Skeletal muscle**	**Macrophage**
KLF2	↑Steatosis (*Cd36*)	Myotube fusion (*Npnt1*)	↓M1 activation
KLF3		?Differentiation (*Mck*)	
KLF4		Myotube fusion (*Npnt1*)	↑M2 activation
KLF5		Differentiation/regeneration Fatty acid oxidation (*Cpt1b, Ucp3*)	
KLF6	Liver development ↑Fibrosis (*Tgfb1, Tgfbr1* in SCs) ↑Steatosis (PPARα) ↑Glucose disposal (*Gck*)		↑M1 activation
KLF7		Quiescence of SCs (*Cdkn1*)	
KLF10		↓Myogenesis	
KLF14	↑Reverse cholesterol transport (*Aopa1*)		
KLF15	↑Gluconeogenesis ↑Amino acid catabolism ↓Lipogenesis (*Srebf1*)	↑Amino acid catabolism ↑Fatty acid oxidation	

*The physiological and pathological functions of KLF members are listed, as are their major targets. In the liver column, the indicated targets are regulated in hepatocytes, except Tgfb1 and Tgfbr1, which are regulated in satellite cells (SCs). KLF3 is suggested to control muscle differentiation, thought this has not been formally tested*.

In addition to their function in metabolic regulation, KLFs may also contribute to metabolic diseases by affecting a variety of other biological processes. For instance, the regulation of inflammation by KLFs via macrophage activation likely contributes to metabolic diseases. In liver inflammation, control of stellate cell activation by KLF6 is important for fibrosis. In addition, regulation of muscle stem cell maintenance and differentiation by KLFs may contribute to sarcopenia and pathological muscle wasting. As such, studies of the cell-specific functions of KLFs would be important for elucidating how KLFs coordinately regulate various cells to maintain tissue and metabolic homeostasis, and how such regulation is involved in metabolic disease development.

Alterations in KLF-dependent regulation of metabolic organs such as the liver, skeletal muscle and adipose tissue likely contribute to the development of cardiovascular diseases via multiple pathways, including insulin resistance, dyslipidemia, and proinflammatory cytokine production within adipose tissue. It is also very likely that KLF-mediated regulation of metabolism within cardiovascular cells is crucially involved in cardiovascular disease development. For instance, KLF5 and KLF15 have been shown to control lipid metabolism within cardiomyocytes, which is essential for maintenance of cardiac homeostasis, at least in part by interacting with nuclear receptors (94, 108, 109). Recent studies have also revealed the crucial involvement of endothelial metabolism in physiological and pathological angiogenesis (110). KLFs may also control endothelial and SMC function through metabolic regulation. This remains to be tested, however.

In this article, we presented an overview of the involvement of KLFs in the hepatic inflammation and fibrosis that leads to NASH. Although we did not cover it in this review, it has been suggested that inflammation contributes to the muscle atrophy seen in both type 2 diabetes and sarcopenia (95, 111). KLFs are also likely involved in muscle inflammation, but their specific functions remain largely unknown (77). Another important unexplored aspect of KLFs is their actions at the crossroad of metabolism and immunity (immunometabolism) (112, 113). It is becoming increasingly clear that the regulatory pathways governing metabolism and inflammation are tightly linked. For instance, on the one hand visceral obesity induces chronic inflammation within visceral adipose tissue, which in turn contributes to the development of cardiometabolic diseases, such as atherosclerosis and type 2 diabetes, in part by promoting inflammation in the affected tissues. On the other hand, regulation of cellular metabolism is integral to the inflammatory and regulatory activation of immune cells (114). It has not yet been well-addressed, but systemic and/or local metabolic disturbances may alter immune cell activities by modulating their cellular metabolism (113, 114). Metabolism and immunity are thus intricately connected at the cell, tissue and system levels. Future studies will need to address how KLF-dependent regulation of metabolism in various cells types, including immune cells, contribute to inflammatory processes in cardiometabolic tissues.

**Figure 4:**
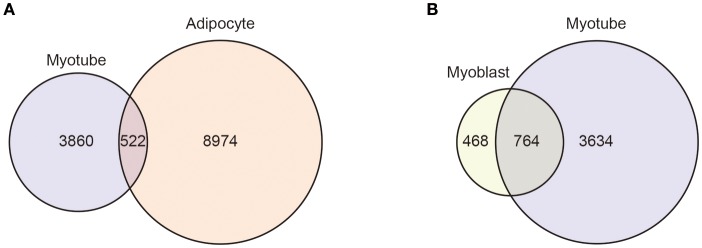
Context-dependent differential KLF5 binding. Venn diagrams showing the overlap between the KLF5 peaks in C2C12 myotubes and 3T3-L1 adipocytes **(A)** and in C2C12 myoblasts and myotubes **(B)**. KLF5 binding peaks were detected using published ChIP-sequencing data (GSE80812 and GSE56872). For myotube peaks, KLF5 ChIP-sequencing of C2C12 cells differentiated for 5 days was used (77). 3T3-L1 cells were treated with an adipocyte differentiation cocktail for 4 h and used in an early differentiated state (115). Reads were mapped to the mm9 genome using STAR (116). Peak calling and annotation was performed using HOMER version 4 (117). Peaks that overlapped blacklisted regions (ENCODE Project Consortium, 2012) or simple repeat regions were removed. Homer-identified peaks with scores ≥ 20 were used as high confidence binding sites (77).

An important functional characteristic of many KLF members, which requires further study, is that their target genes can be different in different cellular and environmental contexts. For instance, ChIP-sequencing showed that KLF5 binding sites in C2C12 myotubes and 3T3-L1 adipocytes differ, and only a small fraction of sites are common between the two cell types (Figure 4A). Moreover, even in the same C2C12 cells, differentiation alters KLF5 bindings sites (Figure 4B) (77). Likewise, KLFs may control different sets of genes in the same cells in response to different microenvironments. An example is that renal injury reduces homeostatic KLF5 binding to the *Cdh1* promoter, and instead KLF5 is recruited to the *S100a8* and *S100a9* promoters in renal collecting duct epithelial cells (118). Furthermore, even at the same promoter, post-translational modifications may modulate KLF function. For example, SUMOylation switches KLF5 function from an activator to a repressor (93). Such context-dependent changes in KLF functions need to be further analyzed, particularly in pathological settings. In that regard, because recent ChIP-sequencing experiments showed that KLF members bind to thousands of genomic sites, it will be important to analyze context-dependent changes in genome-wide binding of KLFs. Currently, ChIP-sequencing data are available for only a few KLFs in limited cell types. In addition, for most KLF bindings sites, it remains unclear whether KLF binding has regulatory importance (e.g., enhancer activity). Genome-wide interrogation of enhancer activation states and the binding of other transcription factors along with transcriptomic analysis, including enhancer RNAs, will provide us with a better understanding of the regulatory functions of KLF members. Another important issue is how the genome-wide target genes of KLF control biological processes as a network. Although studies have so far identified a small number of key targets of KLFs, it is very likely that the target genes that can be identified through ChIP-sequencing and other genome-wide technologies will also play important roles.

As we have discussed in this review, KLFs control a variety of biological processes within metabolic tissues. Although each KLF member appears to have a distinct set of functions, they may also cooperate and/or compete in control of the same process. For instance, KLF5 and KLF15 regulate lipid metabolism in skeletal and cardiac muscle, and KLF6 and KLF15 regulate glucose metabolism in the liver. KLF2/4/6 are involved in macrophage activation. Future studies will need to address how multiple KLF members coordinate metabolism and inflammation. Together, findings from transcriptomics, epigenomics and metabolomics studies may reveal the potential interlinks between KLFs.

Because of their crucial involvement in cardiometabolic diseases, extensive efforts have been made to develop pharmacological agents with which to modulate KLF function. Metformin and perhexiline are two mentioned in this review. Up to now, compounds reported to alter KLF function have acted on pathways affecting KLF expression and post-translational modification, degradation, and transcriptional activity. However, the interactions between KLFs and nuclear receptors are a particularly attractive target in the context of cardiometabolic diseases, as KLFs interact with nuclear receptors to control cellular metabolism and inflammation (119). For instance, we previously showed that KLF5, which contributes to the control of lipid metabolism within cardiomyocytes, interacts with RARα and PPARδ, and its activity can be modulated by ligands for those nuclear receptors (93, 120). This supports the idea that KLFs can be therapeutically targeted using nuclear receptor ligands.

In conclusion, KLFs are important contributors to both homeostasis and pathology in metabolic tissue. They also control metabolism in both cardiovascular and immune cells, as well as immune cell activation. These diverse functions of KLFs may converge at the development of cardiometabolic disease via complex interplay at the cellular, tissue and systemic levels. A better understanding of the KLF-regulated networks that control communications between cells, tissues, and systems could promote development of drugs targeting these disease pathways.

## Author contributions

All authors listed have made a substantial, direct and intellectual contribution to the work, and approved it for publication.

### Conflict of interest statement

The authors declare that the research was conducted in the absence of any commercial or financial relationships that could be construed as a potential conflict of interest.
